# TAMpepK Suppresses Metastasis through the Elimination of M2-Like Tumor-Associated Macrophages in Triple-Negative Breast Cancer

**DOI:** 10.3390/ijms23042157

**Published:** 2022-02-15

**Authors:** Chanju Lee, Soyoung Kim, Chanmi Jeong, Inhee Cho, Juyeon Jo, Ik-Hwan Han, Hyunsu Bae

**Affiliations:** 1 Department of Physiology, College of Korean Medicine, Kyung Hee University, 26 Kyungheedae-ro, Dongdaemoon-gu, Seoul 02447, Korea; lcj8078@naver.com; 2 Cancer Immunology Branch, National Cancer Center, 323 Ilsan-ro, Ilsandong-gu, Goyang 10408, Korea; 3 Department of Science in Korean Medicine, College of Korean Medicine, Kyung Hee University, 26 Kyungheedae-ro, Dongdaemoon-gu, Seoul 02447, Korea; samanda0@nate.com (S.K.); wjdcksal34@naver.com (C.J.); 4 Department of Korean Medicine, College of Korean Medicine, Graduate School, Kyung Hee University, 26 Kyungheedae-ro, Dongdaemoon-gu, Seoul 02447, Korea; okoriental@naver.com (I.C.); leaves-leaf2@hanmail.net (J.J.)

**Keywords:** breast cancer, metastasis, M2-like TAMs, peptide drug conjugate, cancer immunotherapy

## Abstract

Triple-negative breast cancer (TNBC) accounts for approximately 10–15% of all breast cancer cases and is characterized by high invasiveness, high metastatic potential, relapse proneness, and poor prognosis. M2-like tumor-associated macrophages (TAMs) contribute to tumorigenesis and are promising targets for inhibiting breast cancer metastasis. Therefore, we investigated whether melittin-conjugated pro-apoptotic peptide (TAMpepK) exerts therapeutic effects on breast cancer metastasis by targeting M2-like TAMs. TAMpepK is composed of M2-like TAM binding peptide (TAMpep) and pro-apoptotic peptide d(KLAKLAK)_2_ (dKLA). A metastatic mouse model was constructed by injecting 4T1-luc2 cells either orthotopically or via tail vein injection, and tumor burden was quantified using a bioluminescence in vivo imaging system. We found that TAMpepK suppressed lung and lymph node metastases of breast cancer by eliminating M2-like TAMs without affecting the viability of M1-like macrophages and resident macrophages in the orthotopic model. Furthermore, TAMpepK reduced pulmonary seeding and the colonization of tumor cells in the tail vein injection model. The number of CD8^+^ T cells in contact with TAMs was significantly decreased in tumor nodules treated with TAMpepK, resulting in the functional activation of cytotoxic CD8^+^ T cells. Taken together, our findings suggest that TAMpepK could be a novel therapeutic agent for the inhibition of breast cancer metastasis by targeting M2-like TAMs.

## 1. Introduction

Triple-negative breast cancer (TNBC), which accounts for approximately 10–15% of all breast cancers, refers to a specific subtype that tests negative for estrogen receptors (ER), progesterone receptors (PR), and human epidermal growth factor receptor 2 (HER2) protein [1]. The clinical features of TNBC include high invasiveness, high metastatic potential, proneness to relapse, and poor prognosis [2]. The average time to relapse in non-TNBC patients is 35–67 months, whereas that in TNBC patients is only 19–40 months. The mortality rate of TNBC patients within 3 months of recurrence is as high as 75% [3,4]. Owing to its special molecular phenotype, TNBC is not sensitive to endocrine therapy or molecular-targeted therapy. Therefore, chemotherapy is the main systemic treatment, but the efficacy of conventional postoperative adjuvant chemoradiotherapy is poor [5]. Recent studies have reported that the tumor microenvironment, composed of cancer-associated fibroblasts and immune cells, plays an important role in the development and treatment resistance of TNBC [6]. Therefore, there is an urgent need to develop novel treatment regimens and targets.

Metastasis occurs due to the intravasation, migration, extravasation, and colonization of tumor cells. When the primary tumor site becomes hypoxic owing to the excessive proliferation of tumor cells, tumor-derived cytokines, growth factors, and exosomes are released into the tumor microenvironment (TME). These molecules infiltrate the secondary site by continual secretion from the primary tumor, and immune cells are recruited to the secondary site, leading to a tumor-suppressive environment. Among immune cells, recruited macrophages, known as tumor-associated macrophages (TAMs), are key regulators of lung metastasis in breast cancer [7,8], whereas alveolar macrophages are not involved in metastasis [9].

Macrophages are classified as M1-like and M2-like phenotypes. Pro-inflammatory M1-like macrophages release various cytokines and chemokines, such as tumor necrosis factor (TNF)-α, interleukin (IL)-1β, IL-2, IL-6, IL-8, IL-12, IL-23, interferon (IFN)-γ, and CXCL10, which play major roles in inflammation, immunostimulation, and anticancer activity. In contrast, anti-inflammatory M2-like macrophages express various cytokines, chemokines, and proteins, such as IL-10, CCL5, CCL17, CCL18, CCL22, CD206, Arg1, epidermal growth factor (EGF), CD163, and matrix metalloproteinase 9 (MMP-9), which play essential roles in tissue repair, matrix remodeling, immunosuppression, and angiogenesis [10,11]. In the TME, TAMs are thought to have M2-like phenotypes that lead to tumorigenicity and a metastatic state by directly increasing epithelial–mesenchymal transition, extracellular matrix remodeling, and angiogenesis [12,13]. Furthermore, M2-like macrophages have been demonstrated to cause dysregulation of T cell receptor (TCR) signaling and subsequently induce CD8^+^ T cell unresponsiveness [14]. Thus, eliminating M2-like TAMs is a promising strategy to inhibit metastasis.

Recently, many researchers have developed various peptide drug conjugates (PDCs) as novel therapeutic strategies for the treatment of TNBC [15,16]. In recent years, the research community has acknowledged the many advantages of peptides over small molecules and biologics. These include a simpler design, the ability to interact with underexplored targets, cheaper synthesis, decreased immunogenicity, and enhanced tissue penetration. The peptide market is lucrative, as it was estimated to be worth GBP 11–16 billion annually by 2019 [17,18]. However, naturally occurring peptides are often not directly suitable for use as convenient therapeutics because of their intrinsic weaknesses, including poor chemical and physical stability and a short circulating plasma half-life. These aspects must be addressed for their use as medicines [19].

In our previous study, we synthesized a PDC named TAMpepK, consisting of melittin (TAMpep), which binds preferentially to M2-like TAMs [20], a short peptide bridge GGGGS, and a pro-apoptotic peptide d(KLAKLAK)_2_ (dKLA), which induces mitochondrial membrane disruption when internalized into cells [21]. We further reported that this peptide has an M2-like TAM-targeting property in a lung cancer mouse model and verified its anti-angiogenic effect, demonstrating its anti-metastatic potential [21]. Here, we aimed to demonstrate whether TAMpepK selectively eliminates M2-like TAMs in breast cancer metastasis and possesses anti-metastatic potential.

## 2. Results

### 2.1. Inhibition of Breast Cancer Metastasis by TAMpepK in the Orthotopic Model

**Figure 1:**
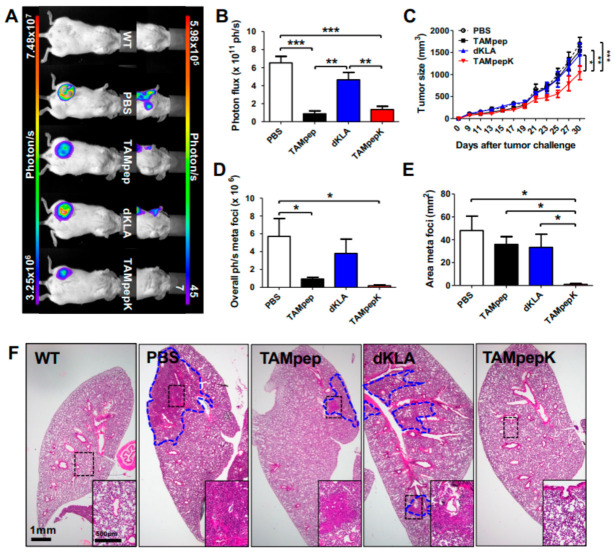
Inhibition of breast cancer metastasis by TAMpepK in the orthotopic model. (**A**) Representative images of bioluminescence emission from upper-body (right panel) and whole-body detection (left panel). (**B**) Quantification of bioluminescence (photon flux/sec) from 4th mammary gland primary tumors and (**C**) caliper measurements of tumor outgrowth. (**D**) Quantification of bioluminescence from half-body detection and (**E**) total area of metastatic site. Data are shown as means ± SEMs; * *p* < 0.05, ** *p* < 0.01, *** *p* < 0.0001. (**F**) Representative images of H&E-stained main lungs showing inhibition of tumor invasion of TAMpepK. Tumor area was guided by blue lines and black-lined boxes were magnified. Total magnification, 1.5× (size bar: 1mm) and 4× (insets; size bar: 500 μm).

The hybrid peptide TAMpepK developed in our previous study [22] showed a strong antitumor effect in lung-cancer-bearing mice, which was found to be mediated by the specific mitochondrial death of M2-like TAMs. We also confirmed that the anti-angiogenic effect of TAMpepK is highly associated with metastasis [22]. In this study, we further verified whether TAMpepK could suppress invasive tumor metastasis and primary tumor growth. First, we generated an orthotopic metastasis model by injecting 4T1-Luc2 cells into the fourth mammary fat pad of NOD-SCID mice, since firefly luciferase reporters could hamper tumor growth and metastasis in the orthotopic model due to an anti-reporter immune response [23,24]. Although NOD-SCID mice are among the most immunodeficient mouse strains with the suppressed function of T, B, and natural killer cells, they have been used in various studies associated with macrophage function, as they harbor macrophages [25]. Here, the wild-type (WT) group was only administered the Matrigel mixture without tumor cells, whereas the other groups were tumor-challenged with 4T1-Luc2 injection. Four weeks after the tumor challenge, half- or whole-body imaging was performed to determine the photon intensity (photons/s) in the primary tumor, lungs, and lymph nodes (LNs) using an in vivo bioimaging system (Figure 1A). The PBS and dKLA groups showed severe tumor progression and distant organ metastasis (meta: *n* = 5 out of 5 mice). The TAMpep group demonstrated significantly decreased photon intensity but exhibited a high metastasis rate (meta: *n* = 4 out of 5 mice). The TAMpepK group also demonstrated inhibition of primary tumor growth. No or a weak signal was detected in the LNs of the TAMpepK group mice (meta: *n* = 2 out of 5 mice). Analysis of the photon intensities and measurement of tumor outgrowth showed that TAMpepK treatment significantly reduced primary tumor growth (Figure 1B,C). The area and photon intensity of the metastatic nodes also significantly decreased after TAMpepK treatment (Figure 1D,E).

Next, H&E staining of the main lungs was performed to confirm metastatic progression, which was hardly detected by in vivo imaging owing to a weak signal (Figure 1F). Unlike lung tissues in the WT mice group, those in the PBS group showed widespread colonies, indicating tumor metastasis, invasion, and outgrowth. The lung tissues in the dKLA and TAMpep mice groups also showed a metastatic burden of 4T1 cells. However, the TAMpepK group showed no pulmonary colonies, even though LN metastasis was detected by bioluminescence imaging, as shown in Figure 1A. These data indicated that TAMpepK was therapeutically effective against breast cancer metastasis in this orthotopic model.

### 2.2. Elimination of M2-Like TAMs by TAMpepK in the Primary Tumor and Lymph Node of the Orthotopic Model

**Figure 2:**
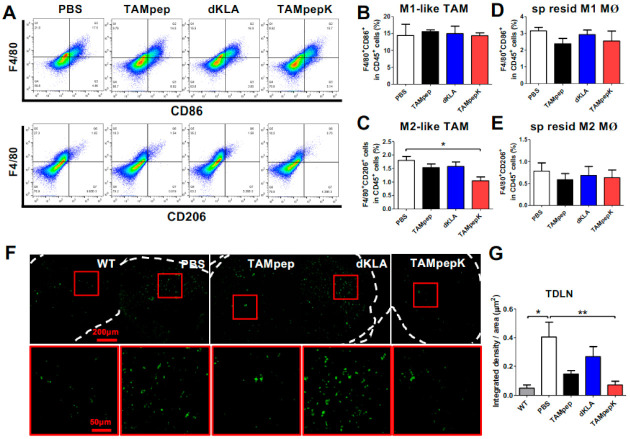
Elimination of M2-like TAMs by TAMpepK in the primary tumor and LN of the orthotopic model. (**A**) M1-like TAMs were marked as F4/80^+^CD86^+^ (upper panel) and M2-like TAMs were plotted as F4/80^+^CD206^+^ (bottom panel) in CD45^+^ cells within 7AAD^neg^ live cells. (**B**–**E**) Percentages of (**B**) M1- or (**C**) M2-like TAMs in CD45^+^ total leukocytes from primary tumor stroma and percentages of (**D**) M1- or (**E**) M2-like resident macrophages in CD45^+^ total leukocytes from spleen tissue. The values are presented as means ± SEMs; * *p* < 0.05 versus the PBS group. (**F**) Representative images of CD206 immunofluorescence staining (green) showing a decrease in M2-like TAMs by TAMpepK compared to PBS, dKLA, and TAMpep groups. Lymph node borders were guided by white dotted lines and red-lined boxes were magnified. Total magnification, 10× (upper panel; size bar: 200 μm) and 40× (lower panel; size bar: 50 μm). (**G**) Fluorescence was quantified as integrated density per area (μm^2^) by Image J. * *p* < 0.05, ** *p* < 0.01.

We further tested the targeting properties of TAMpepK in an orthotopic breast cancer model. Primary tumors were dissociated into single cells and stained with 7AAD, CD45, F4/80, CD86, and CD206. F4/80^+^CD86^+^ (M1-like TAMs) and F4/80^+^CD206^+^ (M2-like TAMs) cells in CD45^+^ leukocytes were plotted after removing the dead cells by gating the 7AAD-negative population (Figure 2A). Only the TMApepK group showed a significant reduction in the live M2-like TAM population compared to the PBS group, whereas there was no change in the percentage of M1-like TAMs (Figure 2B,C). Other peptide treatments, such as dKLA and TAMpep, did not affect the tumor-infiltrated M1- or M2-like TAM populations. Tissue-resident macrophages in the spleen were not affected by TAMpepK treatment, suggesting that TAMpepK specifically targets M2-like TAMs in the tumor stroma (Figure 2D,E).

As M2-like TAMs in distant LNs are highly associated with the early metastatic stage [26], we further verified the presence of M2-like TAMs in tumor-draining LNs (TDLNs) using CD206 staining (Figure 2F,G). Mice in the WT group showed a few macrophages in TDLN, but those in the PBS and dKLA groups exhibited highly increased numbers of M2-like TAMs and enlarged and activated cell bodies. Mice in the TAMPEP group demonstrated lower numbers of M2-like TAMs compared to those in the PBS group, but this difference was not significant. TAMpepK led to the significant elimination of M2-like TAMs in TDLNs. These data demonstrate that TAMpepK reduced pulmonary tumor colonization by eliminating M2-like pro-metastatic TAMs not only from the tumor stroma, but also from the TDLNs.

### 2.3. Inhibition of Metastatic Pulmonary Colonization by TAMpepK in the 4T1-Luc2 Breast Cancer Tail Vein Injection Model

**Figure 3:**
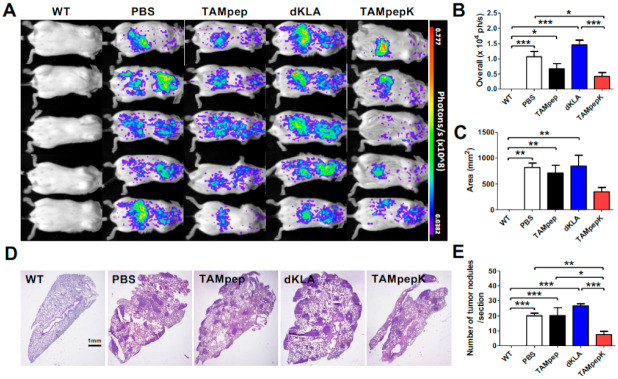
Inhibition of metastatic colonization by TAMpepK treatment in tail vein injection model. (**A**) Images of bioluminescence emission from 4T1-luc2 TV-injected mice. (**B**) Quantification of bioluminescence (photon flux/sec) and (**C**) total area of tumor colonization. (**D**) Representative images of H&E-stained main lungs showing tumor nodules, and (**E**) the number of nodules in the lung was counted. All data represented as the means ± SEMs; * *p* < 0.05, ** *p* < 0.01, *** *p* < 0.0001.

Next, we used a tail vein injection model to investigate the inhibitory effect of TAMpepK on metastatic seeding and colonization. Female BALB/c mice were administered 4T1-Luc2 breast cancer cells (1 × 10^5^ cells) or vehicle PBS via the tail vein. Fifteen days after the tumor challenge, metastatic tumor cell density was determined by bioluminescence imaging (Figure 3A–C). There was no difference in the metastatic area among the PBS, dKLA, and TAMpep groups; however, a marked decline in the metastatic area was observed in the TAMpepK group. Furthermore, photon intensity markedly decreased with TAMpepK treatment compared to that with PBS. H&E staining revealed that the number of pulmonary metastatic lesions in the TAMpepK group was significantly lower than that in the PBS and dKLA groups, whereas TAMpep failed to inhibit colony formation in mice (Figure 3D,E).

### 2.4. Depletion of Metastatic M2 TAMs and Related Genes by TAMpepK in the Tail Vein Injection Model

**Figure 4:**
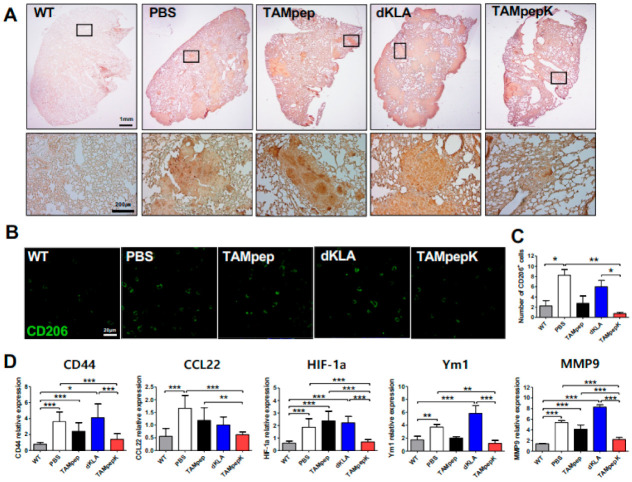
Reduction of metastatic M2 TAMs and related genes by TAMpepK treatment in tail vein injection model. (**A**) Representative IHC staining of CD11b^+^ macrophages and (**B**) CD206^+^ M2-like macrophages in lungs from WT and tumor-challenged mice. (**C**) The number of CD206^+^ cells was counted in 5 random lesions. (**D**) qPCR of metastatic markers CD44, CCL22, HIF-1a, Ym1, and MMP9 in lungs from WT and tumor-challenged mice treated with peptides, respectively. Fold increase of each gene was normalized against the WT group. All data represented as the means ± SEMs; * *p* < 0.05, ** *p* < 0.01, *** *p* < 0.0001.

To confirm whether TAMpepK eliminated M2-like TAMs from tumor-colonized lungs, CD11b^+^ macrophages were analyzed by immunohistochemistry. The results showed the dense infiltration of CD11b^+^ macrophages within tumor nodules in PBS or dKLA mice. However, mice in the TAMpep and TAMpepK groups demonstrated significantly reduced CD11b^+^ macrophages within tumor nodules (Figure 4A). Furthermore, confocal imaging revealed that the reduced macrophage population within the tumor colonies after TAMpepK treatment was associated with the elimination of CD206^+^ TAMs (Figure 4B,C). Indeed, the number of CD206^+^ cells was higher in the lungs of the PBS and dKLA group mice compared to that in the WT group mice. TAMpepK treatment successfully eliminated CD206^+^ M2-like TAMs, leading to a significantly lower CD206^+^ cell count compared with TAMpep treatment (Figure 2). The expression levels of metastasis-related genes were quantified by real-time PCR of lung tissues (Figure 4D). M2 phenotypic markers, such as CCL22, HIF-1a, Ym1, and MMP-9, which are involved in immunosuppression, angiogenesis, epithelial–mesenchymal transition, and invasion [27,28], were significantly upregulated by tumor challenge compared to the WT group. The expression levels of CCL22, HIF-1a, and MMP-9 were significantly lower in the TAMpepK group compared to the PBS group; however, no significant difference was observed in the expression levels of Ym1 between the two groups. *CD44*, the surface receptor regulating cancer cell adhesion critical for colonization and invasion [29], also markedly increased with tumor injection, as shown in the PBS and dKLA groups compared to that in the WT group, whereas this increase was inhibited by TAMpep or TAMpepK treatment. Taken together, TAMpepK blocked tumor colonization in the lungs by targeting M2-like TAMs, resulting in a decrease in metastatic gene expression.

### 2.5. Depletion of CD8^+^ T Cells Related to TAMs by TAMpepK in the Tumor Stroma

**Figure 5:**
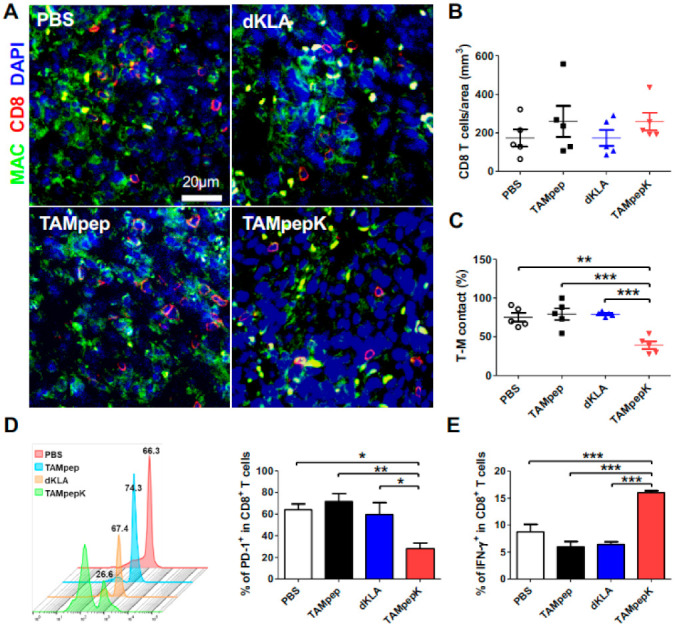
Reduction of CD8^+^ T cells related to TAMs by TAMpepK in the tumor stroma. (**A**) Confocal images of a TV lung slice stained for TAM (CD68^+^; green), CD8^+^ T cells (CD8^+^; red), and nucleus (DAPI; blue). (**B**) Infiltration of CD8 T cells in tumor nodules and (**C**) frequency of CD8^+^ T cells in contact with TAMs was calculated by counting T cells or without TAM contact in each microscopic field of 5 random regions. (**D**) PD-1 expression as exhaustion marker of CD8^+^ T cells in tumor tissue. (**E**) IFN- γ expression as activation marker of CD8^+^ T cells in tumor tissue. All values are the mean and error bars represent SEM. * *p* < 0.05, ** *p* < 0.01, *** *p* < 0.0001.

To further confirm whether the anti-metastatic effect of TAMpepK is related to the infiltration of cytotoxic CD8^+^ T cells (the key players that fight against cancer) into tumor colonies, we investigated the correlation between TAMs and CD8^+^ T cells in pulmonary nodules in tail vein injection cancer-challenged mouse lungs using immunofluorescence staining (Figure 5A). Unexpectedly, we found that TAMs were not responsible for the exact number of CD8^+^ T cells infiltrating the tumor colonies. Depletion of M2-like TAMs with TAMpepK treatment did not alter CD8^+^ T cell counts in the tumor colonies (Figure 5B). Importantly, we observed that most CD8^+^ T cells were surrounded by or in direct contact with abundant TAMs in the PBS, dKLA, and TAMpep groups, but free T cells significantly increased with TAMpepK treatment (Figure 5C). In addition, we determined whether TAMpepK induces the activation of CD8^+^ T cells by the elimination of M2-like TAMs. PD-1 expression as an exhaustion marker in CD8^+^ T cells was significantly decreased in the TAMpepK group compared to other groups (Figure 5D). IFN-**γ** expression as an activation marker in CD8^+^ T cells was significantly increased by TAMpepK (Figure 5E). Given that TAMs reduce the motility of CD8^+^ T cells in the tumor stroma [30], these results suggest that TAMs might directly deactivate and limit the function of CD8^+^ T cells in the tumor. Therefore, our findings suggest that TAMpepK can induce the activation of CD8 T cells by targeting M2-like TAMs.

## 3. Discussion

Here, we used a pro-apoptotic peptide (TAMpepK) to target M2-like TAMs in 4T1 breast cancer metastasis mouse models. As TAMpep has been previously reported to have significant antitumor effects with a high binding affinity for M2-like TAMs [20], we sought to improve the antitumor activity of TAMpep by preparing TAMpepK for an enhanced targeting effect. We have previously demonstrated that TAMpepK inhibits tumor growth and vessel formation in a Lewis lung carcinoma mouse model, suggesting the possibility of suppressing angiogenesis and metastasis [22]. Based on this, we verified the inhibitory effect of TAMpepK on invasive breast cancer and metastasis and observed an M2-targeting effect not only in the primary tumor stroma but also in TDLN, without any effect on the resident macrophages, in the orthotopic model.

Breast cancer is the main cause of cancer-related death among women. Triple-negative breast cancer (TNBC), a subtype of breast cancer, has been clinically characterized by the insufficient expression of estrogen receptor, progesterone receptor, and HER2 [28]. It is the most aggressive subtype of breast cancer and accounts for 12–20% of all breast cancer cases [29]. While primary lesions can be surgically removed in most cases, subclinical micrometastasis and chemoresistance make it intractable. Importantly, most patients with metastatic TNBC eventually relapse, even if they undergo treatment at an early stage [30]. Therefore, there is an urgent need to develop novel treatments and targets.

TAMs, known to promote a cancer metastatic phenotype, are enriched in the stroma surrounding the tumor and avascular areas and support epithelial–mesenchymal transition and intravasation in breast cancer mouse models [31,32]. Although the role of TAMs in cancer is not clear [33,34] as they are originally involved in immune defense reactions and pathogen elimination, there is a positive correlation between poor prognosis and TAM density in more than 80% of breast cancer cases, suggesting the pro-tumorigenic function of TAMs in primary tumors [35,36]. In particular, TAMs overexpress growth factors such as vascular endothelial growth factor and EGF, which are highly associated with the metastatic spread of tumor cells in human breast cancer [37]. TAMs at metastatic sites are also critical for the settlement of disseminated tumor cells accumulating before the arrival of tumor cells. Deng et al. reported that the ablation of *Stat3* or *S1pr1* in myeloid cells is related to the M2-like phenotype, and immunosuppression abrogated metastatic colonization, suggesting a significant role of macrophages in the metastatic niche [38]. A study on macrophage deficiency caused by a colony-stimulating factor-1 (*Csf1*) null mutation showed a dramatic decline in metastatic cell seeding in a TV model, which revealed that tumor cell seeding is strongly dependent on CSF-1 expression, which is crucial for macrophage recruitment into the secondary site [9]. Given that the TV model did not show a primary tumor–metastatic lesion interaction but the adherence and colonization of a secondary organ [39], our data demonstrated that TAMs not only promote metastatic growth in the primary tumor, but also seeding in metastatic lesions. Furthermore, the elimination of TAMs by TAMpepK inhibited the metastasis of breast cancer as well as primary tumor growth and metastatic genes, including HIF-1, Ym1, and MMP9.

Successful depletion of M2-like TAMs by TAMpepK also resulted in a marked increase in free cytotoxic CD8^+^ T cells, as shown in Figure 5. The rich infiltration of cytotoxic T cells is often associated with favorable clinical outcomes [40,41]. CD8^+^ T cells exert cytotoxic activity against tumor cells by triggering apoptotic cell death via granule exocytosis involving perforin and granzymes and via the death ligand/receptor system involving Fas ligand, TNF-α, and TNF-related apoptosis-inducing ligand [42]. However, cytotoxic T cells often fail to kill tumor cells because of their poor infiltration and migration efficiency in the tumor and several dysfunctions induced by the immunosuppressive TME. However, in this study, we did not elucidate the role of CD8^+^ T cells in the inhibitory effect of TAMpepK in the TNBC metastasis model. In future studies, it is necessary to investigate whether the activation of CD8+ T cells is induced by the elimination of M2-like TAMs by TAMpepK.

Our data highlight the potential of TAMpepK in the treatment of breast cancer metastasis and its promising use in targeting M2-like TAMs, which play crucial roles in metastatic colonization and T cell deactivation. Thus, we suggest that TAMpepK targeting M2-like TAMs can be used as a novel therapeutic agent for breast cancer metastasis.

## 4. Materials and Methods

### 4.1. Peptides

TAMpep (GIGAVLKVLTTGLPALISWIKRKRQQ), dKLA (d[KLAKLAKKLAKLAK]), and TAMpepK (GIGAVLKVLTTGLPALISWIKRKRQQGGGGS-d[KLAKLAKKLAKLAK]) peptides were synthesized and purified to greater than 95% purity (GenScript Biotech, Piscataway, NJ, USA). The peptides were dissolved in distilled water of 0.1% acetic acid for storage.

### 4.2. Cell Cultures

The murine breast cancer cell line (4T1) was purchased from the American Type Tissue Culture Collection (Manassas, VA, USA), and the 4T1-Luc2 cell line (BW124087) expressing firefly luciferase (RedFluc) was purchased from Perkin-Elmer (Shelton, CT, USA). The cells were maintained in RPMI 1640 (Welgene, Daegu, Korea) supplemented with 10% fetal bovine serum (Welgene, Daegu, Korea) and 1% penicillin and streptomycin (Invitrogen, Waltham, MA, USA) at 37 °C in a humidified 5% CO_2_ incubator. The medium was refreshed every 2–3 days and reseeded at a density of 5 × 10^5^ cells/mL. All cell lines were authenticated by short tandem repeat profiling. 

### 4.3. Experimental Animals

Female BALB/c mice (6 weeks old, 20–22 g) were purchased from DBL (Seoul, Korea), and female NOD-SCID mice (6 weeks old, 20–22 g) were obtained from Koatech (Gyeonggi, Korea). The mice were maintained in a specific pathogen-free environment with a 12 h light/dark cycle and free access to food and water. Nesting sheets were used for enrichment. After the termination of the experiments, all mice were euthanized using 2% isoflurane and cervical dislocation. All animal experiments were approved by the Institutional Animal Care and Use Committee of Kyung Hee University (KHUASP(SE)-18-088 and 18-133).

For the orthotopic model, 5 × 10^5^ 4T1-luc2 cells were suspended in a serum-free medium and mixed with a Matrigel matrix (Corning, Bedford, MA, USA) at a 1:1 ratio, as previously described [43]. Using a 31G needle, a small incision was made in the 4th mammary gland of NOD-SCID mice under anesthesia with 2% isoflurane, and the cell mixture was slowly injected. The incision site was closed with sutures. Five days after tumor implantation, peptides were diluted with phosphate-buffered saline solution (PBS) to a final concentration of 175 nmol/kg and administered intraperitoneally every 3 days, as previously described [22]. The mice were sacrificed 4 weeks after inoculation. For the tail vein injection model, 1 × 10^5^ 4T1 or 4T1-luc2 cells were suspended in 100 μL PBS and injected into the tail vein of BALB/c mice, as previously described [44]. After 3 days, the peptides were injected intraperitoneally every 3 days. The mice were sacrificed on day 15 of the tumor challenge.

### 4.4. Measurement of Tumor Growth

In the orthotopic model, the primary tumor volume was measured every 3 days during the whole experiment using a digital caliper and calculated using the following formula: volume (mm^3^) = [width (mm)]^2^ × length (mm) × 1/2. At the end of the experiments, tumor growth and metastasis were detected using an in vivo bioluminescence imaging system (NightOWL II; Berthold Technologies GmbH, Wildbad, Germany). Fifteen minutes after D-luciferin (BioVision, Milpitas, CA, USA) injection (4 mg per mouse), luminescence signals were detected with an exposure time of 0.1 s and 4 × 4 binning. In the tail vein injection model, bioluminescence imaging was performed with an exposure time of 60 s and a 16 × 16 binning. Photon energy and tumor area were analyzed using IndiGO software (Berthold Technologies GmbH).

### 4.5. Quantitative Real-Time PCR

Total RNA was extracted from the lung tissues using the easy-BLUE Total RNA Extraction Kit (iNtRON Biotechnology, Gyeonggi-do, Korea). cDNA was generated using CycleScript Reverse Transcriptase (Bioneer, Daejeon, Korea), following the manufacturer’s instructions. Quantitative real-time PCR was performed using a SensiFAST SYBR No-ROX Kit (Bioline, London, UK) on a CFX Connect Real-Time PCR Detection System (Bio-Rad Laboratories, Hercules, CA, USA). The cDNA synthesis conditions were as follows: 95 °C for 15 s, 55 °C for 10 s, and 72 °C for 10 s. Each reaction was performed in triplicate. The base sequences of the primers used were as follows: CD44: forward, 5′-TGGATCCGAATTAGC TGGA-3′; reverse, 5′-GCTTTTTCTTCTGCCCACA-3′. CCL22: forward, 5′-TCCCAGGGGAAGGAATAAA-3′; reverse, 5′-GGTTTGGATCAA GCCCTTT-3′. HIF-1α: forward, 5′-TCCCTTTTTCAAGCAGCAG-3′; reverse, 5′-TGCCTTGTATGGGAGCATT-3′. GAPDH: forward, 5′-CCCAGAAGACTGTGGATGG-3′; reverse, 5′- CACATTGGGGGTAGGAACAC-3′. The housekeeping gene GAPDH was used as an internal control.

### 4.6. Hematoxylin and Eosin (H&E) Staining

Lung tissues were fixed overnight in 10% neutral buffered formalin and cut to 4 μm thickness after embedding in paraffin. The sections were dipped in xylene, followed by a series of ethanol gradations at 100%, 90%, 80%, and 70% and washing under running tap water for rehydration. The nuclei were stained with hematoxylin solution for 10 min. After differentiation by dipping in 1% acid alcohol, eosin–phloxine staining was performed for 15 min to counterstain the cytoplasm. All the steps were performed after washing the sections in running tap water for 5 min. Sections were dehydrated by dipping in 70%, 80%, 90%, and 100% ethanol, respectively, and cleared with xylene. The sections were mounted using DIAMOUNT medium and images were captured using a Nikon Eclipse Ci-L microscope-DS-Fi2 CCD camera (Nikon, Tokyo, Japan).

### 4.7. Immunohistochemistry

Tissue samples were deparaffinized and rehydrated as previously described. The tissue antigen was heat-retrieved using sodium citrate buffer (pH 6.0) for 1 min at 121 °C. The tissue sections were incubated with 3% H_2_O_2_ for 15 min and blocked with 1.5% bovine serum albumin (BSA) containing 0.2% Triton X-100 for 1 h. Slides were incubated overnight at 4 °C with rat anti-mouse CD11b (1:200; Bio-Rad Laboratories) antibodies diluted in 0.5% BSA. After washing with 0.5% BSA, the tissue was incubated with biotinylated anti-rat IgG (1:500; Vector Laboratories, Burlingame, CA, USA) secondary antibodies diluted in 0.5% BSA for 1 h. The VECTASTAIN Elite ABC HRP Kit and DAB Substrate Kit (both from Vector Laboratories) were used for colorimetric detection. All images were captured using a Nikon Eclipse Ci-L microscope-DS-Fi2 CCD camera (Nikon).

### 4.8. Flow Cytometry

Primary tumor tissues or spleens were dissociated using a gentleMACS Dissociator (Miltenyi Biotec GmbH, Bergisch-Gladbach, Germany) after enzyme digestion in serum-free RPMI 1640 medium containing DNase I (1 U/mL; Roche, Basel, Switzerland) and collagenase D (1 mg/mL; Roche) for 20 min at 37 °C with gentle agitation. After single-cell dissociation, the cells were filtered through a 40 μm cell strainer, and the red blood cells were eliminated using BD Pharm Lyse buffer (BD Biosciences, San Jose, CA, USA). The cells were stained by incubating at 4 °C for 1 h using the following antibodies: CD45-FITC, F4/80-PE, CD206-APC, CD86-PE/Cy7, CD8-APC, PD-1-PE, and IFN-γ-PE/Cy7 (e-Bioscience, CA, USA). After staining, the cells were analyzed using a BD FACSLyric flow cytometry system (BD Biosciences) and FlowJo software (Treestar, San Carlos, CA, USA).

### 4.9. Immunofluorescence

All sections were deparaffinized, and antigen retrieval was performed as previously described. For the detection of M2-like TAMs, lymph nodes or lung tissues were blocked with 1.5% BSA containing 0.2% Triton X-100. The tissues were incubated with rat anti-mouse CD206 primary antibodies (1:1000; Bio-Rad Laboratories) and visualized using Alexa-488 conjugated anti-rat IgG (1:1000; Invitrogen). All antibodies were diluted in 0.5% BSA solution. To detect TAMs and CD8^+^ T cells in tumor nodules of lung tissues, the sections were incubated overnight at 4 °C with the following antibodies: rabbit anti-mouse CD8a (1:2000; Abcam, Cambridge, MA, USA) and rat anti-mouse CD68 (1:1000; Bio-Rad Laboratories). They were then visualized after incubating with the following antibodies for 1 h at room temperature: Alexa-594 conjugated anti-rabbit IgG (1:1000; Invitrogen) and Alexa-488 conjugated anti-rat IgG (1:1000; Invitrogen). Five fields of different tumor nodules in the lungs were randomly selected and visualized using an LSM 800 laser scanning confocal microscope (Carl Zeiss, Jena, Germany).

### 4.10. Statistical Analysis

All data are expressed as the mean ± standard error of mean (SEM). To compare the mRNA expression levels between groups, a one-way analysis of variance (ANOVA) followed by the Newman–Keuls test was employed. For others, statistical significance was calculated using one-way ANOVA followed by Tukey’s post-hoc test and Student’s *t*-test. All analyses were performed using Prism software version 5.01 (GraphPad Software, San Diego, CA, USA).

## Data Availability

All data generated or analyzed during this study are included in this published article.
